# Evaluation of the Inhibitory Effects of Bavachinin and Bavachin on Human Monoamine Oxidases A and B

**DOI:** 10.1155/2015/852194

**Published:** 2015-10-19

**Authors:** Najla O. Zarmouh, Elizabeth A. Mazzio, Faisel M. Elshami, Samia S. Messeha, Suresh V. K. Eyunni, Karam F. A. Soliman

**Affiliations:** College of Pharmacy and Pharmaceutical Sciences, Florida A&M University, Tallahassee, FL 32307, USA

## Abstract

Monoamine oxidase B inhibitors (MAO-BIs) are used in the early management of Parkinson's disease (PD). Long-term suspected side effects of MAO-B classical inhibitors established the need for safer alternative therapeutic agents. In our study, the flavanone bavachinin (BNN) and its analog bavachin (BVN) found in the seeds of *Psoralea corylifolia* L. ethanolic extract (PCSEE) were investigated for their *human* MAO-A and MAO-B (*h*MAO-A and *h*MAO-B) inhibition. Both PCSEE and BNN effectively reduced *h*MAO-B activity more than *h*MAO-A while BVN had activating effects. BNN showed selective *h*MAO-B inhibition (IC_50_ ~ 8.82 *μ*M) more than *h*MAO-A (IC_50_2009;~ 189.28 *μ*M). BNN in the crude extract was determined by HPLC, also validated by TLC showing a yield of 0.21% PCSEE dry weight. BNN competitively inhibited *h*MAO-A and *h*MAO-B, with a lower *h*MAO-B *K*_*i*_ than *h*MAO-A *K*_*i*_ by 10.33-fold, and reduced *h*MAO-B *K*_*m*_/*V*_max_ efficiency ratio to be comparable to the standard selegiline. Molecular docking examination of BNN and BVN predicted an indirect role of BNN C7-methoxy group for its higher affinity, selectivity, and reversibility as an MAO-BI. These findings suggest that BNN, which is known to be a potent PPAR-*γ* agonist, is a selective and competitive *h*MAO-B inhibitor and could be used in the management of PD.

## 1. Introduction

Parkinson's disease (PD) is a highly prevalent neurodegenerative disorder in the aging population, especially in developed countries [1]. PD hallmark pathological feature is the major depletion of the neurotransmitter dopamine within the substantia nigra pars compacta. For this reason, therapeutic management of PD relies on sustaining dopaminergic function required for neuromotor control with drugs such as time release SINEMET CR (L-dopa with carbidopa) and monoamine oxidase B inhibitors (MAO-BIs). While L-dopa remains the most efficient agent for symptomatic relief, its gradual loss of efficacy (wearing off) is the rationale for deterring its use as a first line of therapy where alternative therapies such as MAO-BIs are being used [2]. MAO-BIs were recently found to have multiple therapeutic benefits for neuronal degeneration. Besides their ability to prevent age-associated cellular dysfunction [3], MAO-BIs attenuate oxidative damage [4], exert neuronal antiapoptotic effects [5], and inhibit abnormal *γ*-aminobutyric acid (GABA) produced by monoamine oxidase B (MAO-B) in striatum reactive glia [6, 7]. Moreover, rasagiline (RAS) and its metabolites may also provide neuroprotection [8] and inhibit acetylcholinesterase, providing benefit to both PD and Alzheimer's disease (AD) patients [9].

MAOs (EC 1.4.3.4) are oxidizing flavoenzymes that catalyze the oxidative deamination of biological and xenobiotic monoamines within the neurons and the astrocytes [10]. There are two primary isozymes, MAO-A and MAO-B, which share 70% of their sequence identity and the dependence on the covalently linked flavin adenine dinucleotide (FAD) cofactor in their amino acid active sites [11]. However, their active sites have other properties that may be selected for different substrates or targeted by specific inhibitors. Recent reports suggested the ability of both MAOIs, A and B, to attenuate oxidative stress [3, 12]. However, MAO-B is more abundant and more active in the human basal ganglia [13], and the use of MAO-BIs is considered safer than using MAO-AIs for depression.

The side effects and contraindications of MAO-BIs are largely attributable to their irreversibility and their irreversible cross-reactivity with MAO-A. The specific irreversible inhibition of MAO-A may lead to accumulation of peripheral dietary tyramine which can initiate hypertensive crisis, serotonin syndrome [14, 15], or behavioral aggression [16]. Therefore, the use of selective irreversible MAO-AIs remains limited for managing depression [17] or replaced with reversible MAO-A and MAO-B inhibitors [18], which are better suited to treat depressive or cognitive maladies. On the other hand, the only currently FDA-approved selective MAO-BIs are selegiline (DEP) and RAS [19], both of which bind in an irreversible noncompetitive fashion, forming covalent adducts to the FAD cofactor within the MAO-B active site [20–22]. That irreversible inhibition can have certain disadvantages, including low sensitivity to increase the endogenous substrate, dopamine [23], loss of selectivity with repeated administration, and slow and variable enzyme recovery rates following the inhibitor withdrawal [24] as the biosynthesis for* human brain *MAO-B is estimated to be approximately 40 days [25]. In recent clinical trials, antiparkinsonian effects of new safer reversible MAO-BIs, such as safinamide (SAF), delayed the time of starting L-dopa in PD patients [26]. Hence, searching for alternative, effective, and safer reversible MAO-B inhibitory agents is an important area in the pharmaceutical research.

In a continuous search for novel natural MAO-BIs in our laboratory, the ethanolic extract of* Psoralea corylifolia* L. seeds (PCSEE) [27] had shown a potential to potently inhibit* human *MAO-B [28]. The herb seeds investigated are one of the popular ethnobotanicals used in Ayurvedic and Chinese medicine in various diseases including cardiovascular and skin inflammatory diseases [29]. With its unique constituents and properties, it was recently suggested for novel drugs in phytomedicine [30]. Additionally, this plant extract and its constituents showed phytoestrogenic [31], antidepressant [32], neuroprotective [33], anti-inflammatory [34], and antioxidant properties [35] that could be beneficial in neurodegeneration. Here, we are reporting that one of the PCSEE prenylflavanones constituents, bavachinin (BNN), showed competitive MAO-B inhibitory effects while its analog bavachin (BVN) was not effective. Therefore, the current investigation was designed to characterize and understand the mode of inhibition of BNN. Our investigation may provide for newer generation of MAO-B reversible inhibitors for drug therapy in PD and other neurological disorders.

## 2. Materials and Methods

### 2.1. Materials and Preparations

Human* h*MAO-A and* h*MAO-B used were separately derived from BTI-TN-5B1-4 insect cells infected with cDNA containing recombinant baculovirus.* h*MAO isozymes active units (U) were supplied by Sigma-Aldrich (St. Louis, MO) and a final concentration of 0.88 U/mL of each isozyme was used. Separate* h*MAO-A and* h*MAO-B upon purchase were aliquoted with cold 10 mM HEPES in Hank's Balanced Salt Solution (HBSS) (pH 7.4) and kept in −80°C until use. (S)-enantiomer BNN (≥95% pure) and DEP (irreversible MAO-BI) were purchased from Sigma-Aldrich, and (S)-enantiomer BVN was obtained from Santa Cruz Biotechnology Inc. PCS seeds preserved in nitrogen were purchased from East Earth Trade Winds (Redding, California). For PCS ethanolic extraction, the dried fine powder was extracted following our previous method of repeated heat reflux supported maceration procedure. The fine ground dried seeds of 30 g powder were macerated twice with 99.95% ethanol for two days followed by 8 h Soxhlet reflux at 60–70°C. Ethanol was renewed every 2 h for excessive continuous extraction. The combined portions of the ethanol extracts were evaporated in a fume hood for several days to obtain an oily crude extract.

### 2.2. *h*MAO-A and* h*MAO-B Activity Assay

The chemiluminescent assay was used to confirm PCSEE MAO-A and MAO-B inhibitory effects and to test BNN and BVN* h*MAO-A and* h*MAO-B inhibition [36] using MAO-Glo kit (Promega; Madison, WI). Each enzyme's Arbitrary Light Unit (ALU) was measured in the presence of PCSEE, BNN, BVN, and standard DEP as an MAO-BI positive control. Briefly,* h*MAO-A and* h*MAO-B isozymes were diluted to 2x with reaction buffer (pH 7.4) and preincubated with 4x PCSEE, BNN, BVN, or DEP working solutions at RT for 30 min in white opaque 96-well plates. For determining activity inhibition, final 8.5 *μ*g/mL concentrations of PCSEE, BNN, BVN, and DEP were used. For IC_50_ determination, 8x PCSEE and BNN working solutions were serially diluted using reaction buffers (pH 7.4) to make a 4x concentration. Ten points' range of PCSEE (1.0 to 250.0 *μ*g/mL) and BNN (up to 400 *μ*M (135.4 *μ*g/mL)) final concentrations was used. Controls used were with and without ethanol. Ethanol solvent in controls was kept to a maximum final (volume) of ≤2%. Each isozyme was substituted with the reaction buffer for the blank. Based on our preliminary optimizations and Valley's method [36], the reaction was initiated by adding 4x luciferin derivative substrate (LDS) for a final (concentration) of 40 and 4 *μ*M for* h*MAO-A and* h*MAO-B reactions, respectively. The final volume per well of each reaction was 50 *μ*L. The reaction was optimized for the amount of A and B enzyme used to be incubated for less than 3.5 h at RT. To stop the reaction and produce the luminescence signal RLDR was added to all wells, 50 *μ*L to each well, and incubated for a further 30 min. ALU produced was detected by Synergy HTX Multi-Reader (Bio-Tek).

### 2.3. Quantification and Identification of BNN in the Crude Extract

#### 2.3.1. TLC Analysis

Silica gel on thin layer chromatography (TLC) Alu foils with fluorescent indicator 254 nm silica gel matrix (5 *∗* 10 cm^2^) (Fluka Analytical Sigma-Aldrich) was used. An aliquot of about 20 *μ*L of ethanol solution of 20 mg/mL PCSEE was directly deposited as short band onto a 1 cm height from the bottom of the TLC plate. The BNN (S)-enantiomer isoform (Sigma-Aldrich) was dissolved in ethanol and spotted as a second short band. TLC plate was carefully placed and developed in a saturated closed glass developing chamber with a developing solvent system of pure water : ethanol : acetone (5 : 1 : 3 ratio) of 0.5 cm height. Before solvent front reached at most 0.5 cm away from the edge plate was taken out carefully. The developed TLC plates were then allowed to dry before being visualized under UV light at 254 and 366 nm.

#### 2.3.2. HPLC Analysis

BNN quantification in PCSEE was detected by HPLC using a slightly modified separatory technique [37]. HPLC gradient solvents and supplies were purchased from Sigma-Aldrich (St. Louis, MO). Shimadzu HPLC system used was with an SS420X instrument interface docked to a Waters Autosampler Model 717 Plus (Shimadzu Scientific Instruments, Columbia, MD; Waters, Milford, MA, USA). An SPD-20A UV detector at 240 nm was used to detect BNN concentration in PCSEE. Briefly, mobile phase used consisted of filtered and degassed solution of 67% HPLC gradient methanol and 33% of 20 mM ammonium acetate buffer in pure water (pH 4.00). BNN standards serial concentrations and PCSEE samples were diluted in the mobile phase, and the injection volume was set at 25 *μ*L per injection. Flow rate was isocratic at 1 mL/min. The HPLC separation was carried out by a 5 *μ*m 300 A 4.6 × 100 mm C-18 Venusil column ABS (VWR, Radnor, PA, USA).

### 2.4. Enzyme Kinetics

To define the BNN effect on each isozyme's Henri-Michaelis-Menten hyperbolic regression curve and parameters (*K*_*m*_, *V*_max_, and *V*_max_/*K*_*m*_), MAO-Glo Assay was used after preliminarily determining the best incubation time for initial velocity of activity. The assay was carried out under the same conditions in white opaque 96-well plates. Standard deprenyl (DEP) was used as a positive control for the mode of inhibition. We acquired seven LDS data points differing logarithmically, by use of serial dilution of duplicate concentrations. The substrate preparation was carried out at gradual 4x final LDS 150, 75, 37.5, 18.75, 9.38, 4.69, and 2.34 *μ*M and no substrate for* h*MAO-A and 40, 20, 10, 5, 2.5, 1.3, and 0.65 *μ*M and no substrate for* h*MAO-B. Based on IC_50_ value of each enzyme, 4x BNN fixed concentrations were prepared. BNN concentrations were in high quadruplets for* h*MAO-A (18.75, 75.0, and 300 *μ*M) and in low duplicates for* h*MAO-B (10, 20, and 40 *μ*M). Controls without BNN were prepared simultaneously. 2x enzyme concentrations for a constant final (concentration) of 0.88 U/mL were added to BNN for both isoforms and let for 30 min incubation. Initiation of reactions was started by mixing the enzyme and inhibitor (or buffer) to the 4x LDS in the wells. After 1.5 h (for* h*MAO-A assay) and 2 h (for* h*MAO-B assay) of incubation at RT, the initial rate of this reaction was inhibited by doubling the volume of each well with RLDR. Developed lights in all of the inhibited reactions were measured after 30 min by illuminometer of Synergy HTX Multi-Mode Reader (Bio-Tek, Winooski, VT, USA). The data were plotted for Michaelis-Menten equation as a nonlinear curve, which then was transformed and presented as a linear curve for double-reciprocal Lineweaver-Burk plots. *K*_*m*_, *V*_max_, and *V*_max_/*K*_*m*_ values were computed and presented as folds of relative change by Michaelis-Menten equation analysis using GraphPad Prism (GraphPad Prism Software, Inc., CA, USA). Dissociation constant of each isozyme-inhibitor complex (*K*_*i*_) was also calculated as a global share value for each group of data sets, according to the competitive mode of inhibition equation of BNN competitive model or to the noncompetitive model for DEP with* h*MAO-B.

### 2.5. Molecular Modeling and Docking

The X-ray structures of MAO-A and MAO-B Ligand Binding Domain have been developed from the Research Collaborator for Structural Bioinformatics (RCSB) protein databank (PDB) and used for docking purposes. Since the complexes in this study have bound ligands, HYBRID (OEDocking v3.0.1, OpenEye Scientific Software, Santa Fe, NM) was chosen as the appropriate method for our docking study [39]. HYBRID was used to validate the correct bound ligand structure prediction. The docking poses of the bound ligands in the crystal structures of human MAO-A-harmine complex (2Z5X) and human MAO-B-2-(2-benzofuranyl)-2-imidazoline complex (2XCG) were validated. The best ten poses retrieved through redocking were identical to the original poses of the cognate ligands in MAO-A and MAO-B crystal structures with root mean square deviation less than 2 Å indicating the OEDocking applications reliability as a docking tool in our modeling studies.

The compounds in the present study, BNN, BVN, and safinamide (SAF), were sketched using Sybyl sketch Sybyl-X 1.3 Modeling suite (SYBYL-X 1.3, Tripos International, St. Louis, MO). Energy was minimized and stored as a molecule (.sdf) file. The conformer ensembles of these compounds were generated using OMEGA v2.4.6 (OpenEye Scientific Software, Santa Fe, NM) [40] prior to docking to ensure the retaining of low strain energy conformations in the ensemble. By using Structure Preparation tool for both isozymes, Chain A was extracted, hydrogen atoms were added, and potential bumps were corrected. Both isozymes were energy minimized using MMFF94s force fields and charges assigned. The water molecules in and around each MAO isozyme active site were retained. The resulting refined isozymes were used for docking the ligands. Predicted scores of affinity were presented as HYBRID Chemgauss4 scores.

### 2.6. Statistical Analysis

Data analyses were performed by GraphPad Prism 6.02 software. Data were presented as the mean ± SEM, *n* = 3 at least, representing at least two independent experiments unless otherwise indicated. Inhibitory potency was expressed as the mean average of 50% normalized inhibitory concentration (IC_50_ ± SEM) of at least two independent experiments. IC_50_ was obtained by interpolation of logarithmic concentration-inhibition best fit curves for *R* ^2^. Relative selectivity (RS) folds were calculated as the ratio of* h*MAO-A IC_50_/*h*MAO-B IC_50_. Selectivity index (SI) was determined by *K*_*i*_ *h*MAO-A/*K*_*i*_ *h*MAO-B ratio. Significance of difference between the controls versus treatments was determined by using one-way ANOVA followed by Dunnett's or Tukey's multiple comparisons test. Significance of difference between two sets of data was determined using two-way ANOVA followed by Sidak's multiple comparisons test. For illustration and quantification of detected PCSEE and BNN peaks on HPLC, EZSTART (version 7.4) software was used.

## 3. Results

### 3.1. *h*MAO-A and* h*MAO-B Inhibitory Effects of PCSEE, BNN, and BVN

**Figure 1:**
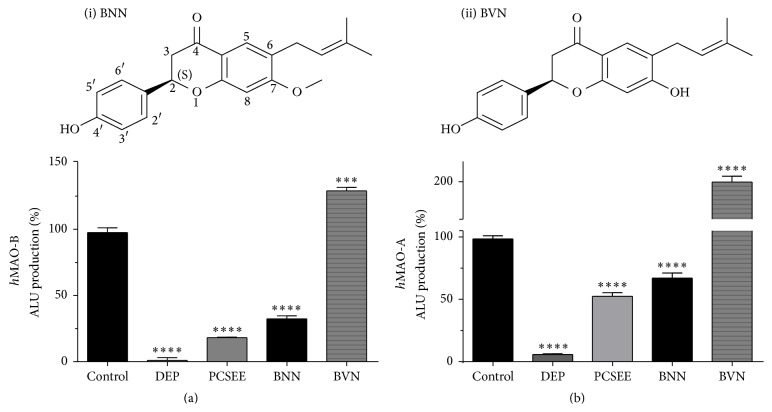
Bavachinin (BNN) and bavachin (BVN) inhibitory efficacy on recombinant* human *monoamine oxidases (*h*MAOs) compared to* Psoralea corylifolia* ethanolic extract (PCSEE) and standard MAO-B inhibitor selegiline (DEP) at 8.5 *μ*g/mL. BNN structure (4′-hydroxy-7-methoxy-6-(3-methyl-2-butenyl) flavanone, 7-O-methylbavachin, mw = 338.4) (i) is a BVN analog with the methyl group at C7-OMe position substituting the hydrogen (ii). BNN and BVN differently affected* h*MAO-B (a) and* h*MAO-A (b). Data points were presented as the mean ± SEM, for at least *n* = 3. The significance of difference between each control versus treatments for each data set was determined using one-way ANOVA followed by Dunnett's multiple comparisons test. ^*∗∗∗*^ *p* < 0.001; ^*∗∗∗∗*^ *p* < 0.0001.

The effect of PCSEE, BNN, and BVN with standard DEP were measured. PCSEE significantly inhibited both* h*MAO-A and* h*MAO-B with more effectiveness in inhibiting* h*MAO-B (Figure 1). Two of its constituents, BNN and its analog BVN, were tested for their potential to inhibit* h*MAO-A and* h*MAO-B (Figures 1(i) and 1(ii)). The data show that BNN displays a highly significant inhibitory effect (*p* < 0.0001) on* h*MAO-B (Figure 1(a)), but, in the presence of* h*MAO-A (Figure 1(b)), BNN inhibitory effect was less. On the contrary, BVN did not show any inhibitory effects on any of the isozymes (Figures 1(a) and 1(b)) and, in contrast, a significant increase of both* h*MAO activity signals was observed particularly with* h*MAO-A (>2-fold).

### 3.2. *h*MAO-A and* h*MAO-B Inhibitory Potency and Selectivity of BNN and PCSEE

**Figure 2:**
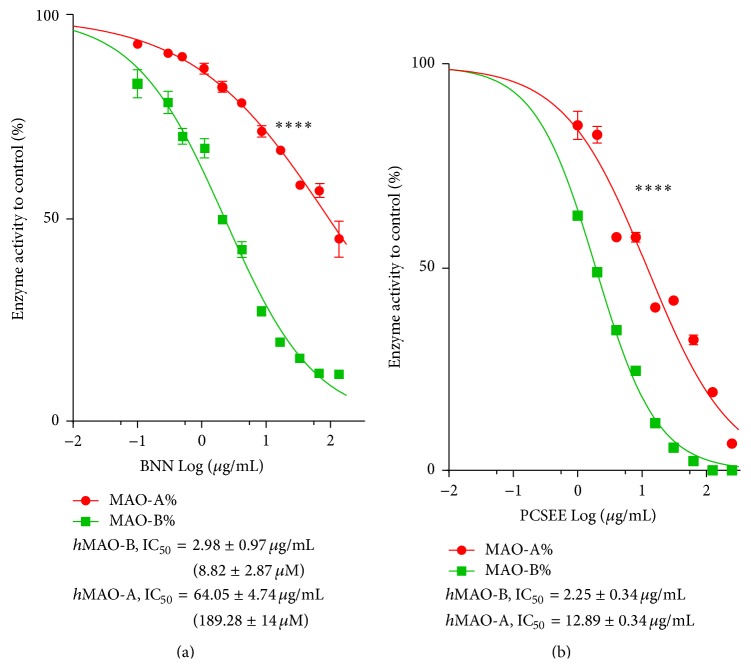
BNN inhibitory potency on recombinant* human *monoamine oxidases isozymes (*h*MAO-A and* h*MAO-B) compared to PCSEE. BNN (a) and PCSEE (b)* h*MAO-B inhibition is more selective than* h*MAO-A isoform inhibition. BNN shows close* h*MAO-B inhibition potency to PCSEE but with higher selectivity to inhibit B than PCSEE. Arbitrary Light Units (ALU) were measured at 25°C. Data points and IC_50_ values were represented by the mean ± SEM, *n* = 3 at least. IC_50_ values were calculated from two separate experiments. Significance of difference between every two data sets was determined using two-way ANOVA followed by Sidak's multiple comparisons test. ^*∗∗∗∗*^ *p* < 0.0001.

BNN inhibitory potency on* h*MAO-A and* h*MAO-B isozymes was compared to PCSEE (Figure 2). Using the luminescence assay, by which DEP IC_50_ values of* h*MAO-A and* h*MAO-B were 14.85 and 0.130 *μ*M, respectively (data not shown), BNN and PCSEE inhibitory effects were dose dependent on both isozymes but with different potencies. In Figure 2(a), BNN showed* h*MAO-B inhibition potency which was significantly higher than* h*MAO-A inhibition (*p* < 0.0001) as it exerted a steeper slope of* h*MAO-B inhibition with an average IC_50_ of 8.82 *μ*M (2.98 *μ*g/mL). BNN showed a gentler* h*MAO-A inhibition slope with an average IC_50_ of 189.28 *μ*M (64.05 *μ*g/mL), pointing out BNN relative selectivity to inhibit* h*MAO-B by an average of 21.46-fold (Figure 2(b)). PCSEE also exerted* h*MAO-A and* h*MAO-B inhibition with more selectivity to inhibit* h*MAO-B (average IC_50_ = 2.25 *μ*g/mL) by 6.26-fold (*h*MAO-A average IC_50_ = 12.89 *μ*g/mL). Although BNN and PCSEE potencies to inhibit MAO-B were similar, BNN was more selective than PCSEE to inhibit MAO-B by 3.41-fold.

### 3.3. Identification of BNN in PCSEE

**Figure 3:**
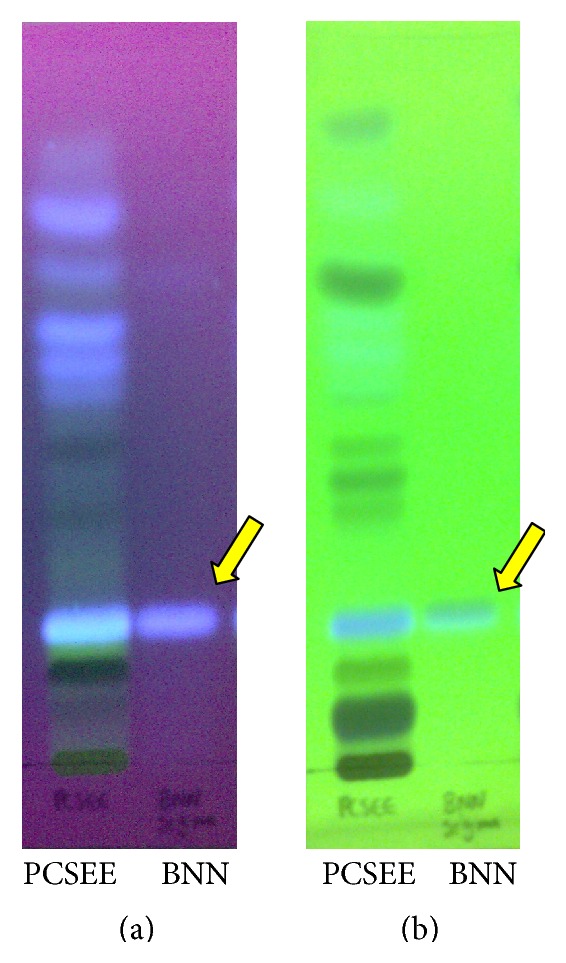
TLC plate's profile of PCSEE (left band) and BNN (right band) visualized (a) under UV 254 nm and (b) under UV 366 nm.

BNN was identified in the PCSEE extract by silica gel TLC (Figure 3). BNN *R*_*f*_ was repeatedly localized at 0.26 with a solvent front of 8 cm on TLC by both UV lights. In Figure 3(a), BNN showed the same fluorescence color as in the matching *R*_*f*_ band of PCSEE under 254 nm UV wave. In Figure 3(b), BNN had similar fluorescence of the matching *R*_*f*_ band of PCSEE under UV light of 366 nm. This chromatographic method highlighted the chemical differences of PCSEE and BNN and presence of BNN as one of its constituents.

### 3.4. Quantification of BNN in PCSEE and PCS

**Figure 4:**
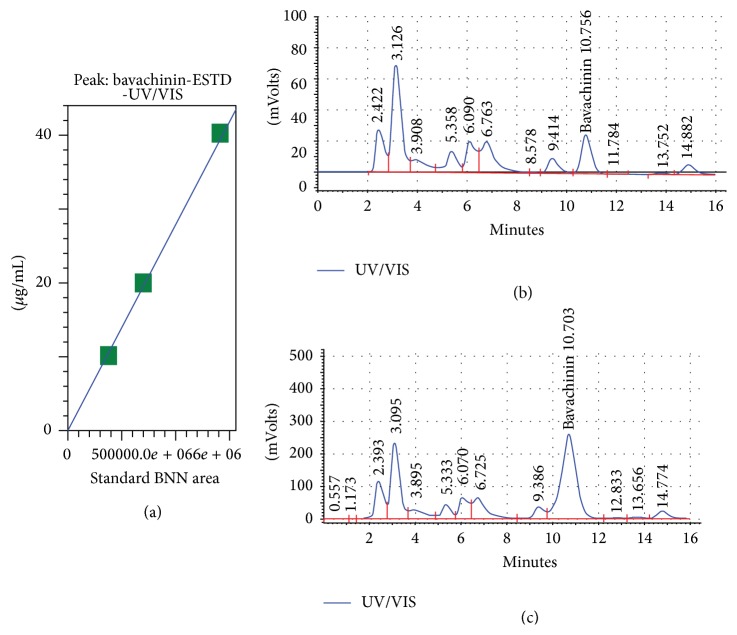
BNN total concentration in PCSEE determined by HPLC at 240 nm using BNN standard (*R* ^2^ = 0.997). (a) BNN detected peak in PCSEE in (b) and spiked PCSEE with BNN standard in (c) confirmed BNN presence at 10.703 min. Crude extract concentration of BNN was approximately 0.210 ± 0.004% of PCSEE. Statistical data were presented as mean ± SEM, *n* = 3.

BNN validation and quantification in PCSEE were detected using HPLC technique (Figure 4). In Figure 4(a), BNN standard was linear with *R* ^2^ of 0.997. BNN detected peak in PCSEE (Figure 4(b)) was also confirmed by spiked PCSEE with BNN standard in Figure 4(c) which resulted in a significant increase at 10.703 min of running time. BNN yield in PCSEE was detected to be 0.210 ± 0.004% w/w dry PCSEE (average 20.96 ± 0.43 *μ*g/mL of BNN in PCSEE solution (10 mg/mL)).

### 3.5. The Mode of Inhibition of* h*MAO-A and* h*MAO-B by BNN

**Figure 5:**
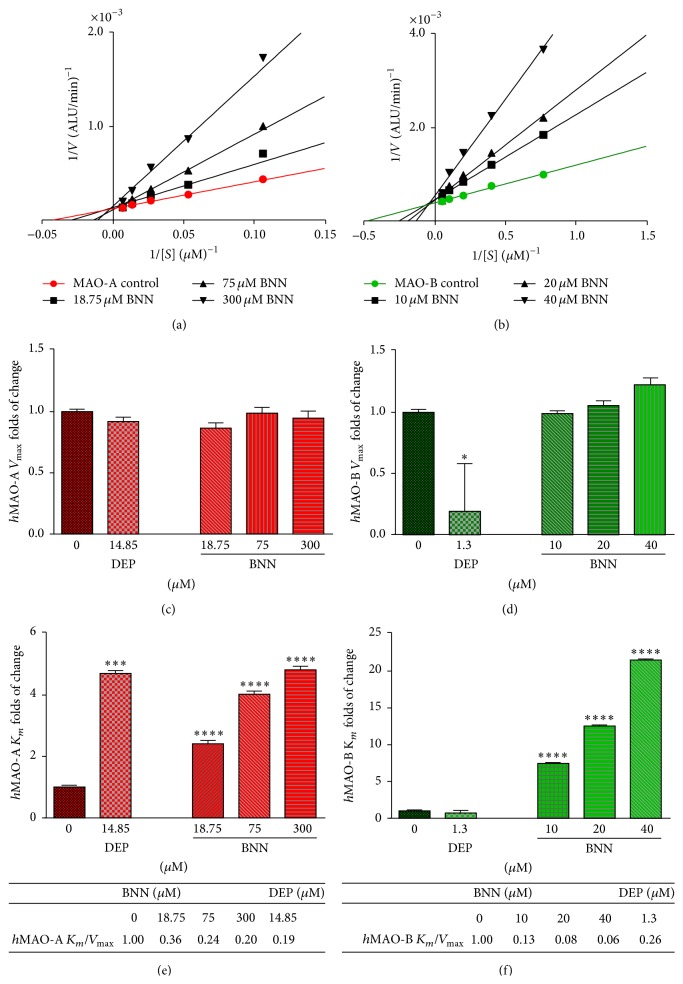
Bavachinin (BNN) effects on* human* monoamine oxidases A and B (*h*MAO-A and* h*MAO-B) kinetics. Illustrating the initial velocity (*V*) with substrate concentrations ([*S*]) with or without BNN as Lineweaver-Burk plots of* h*MAO-A (a) and* h*MAO-B (b). Michaelis-Menten kinetics parameters were presented as folds of change by BNN compared to standard deprenyl (DEP). Parameters are maximum velocity (*V*_max_), Michaelis Constant (*K*_*m*_), and relative *V*_max_/*K*_*m*_ in* h*MAO-A ((c) and (e) with table) and* h*MAO-B ((d) and (f) with table), respectively. Data points were presented as the mean ± SEM, *n* = 3, from the average of four separate experiments for BNN and one experiment for DEP. The significance of the difference between the controls versus inhibitors was determined using one-way ANOVA followed by Dunnett's multiple comparison test. ^*∗*^ *p* ≤ 0.05; ^*∗∗∗*^ *p* < 0.001; ^*∗∗∗∗*^ *p* < 0.0001.

We initially optimized the time of incubation for the examined enzymes to be 1.5 and 2 h incubation for* h*MAO-A and* h*MAO-B activities, respectively. These incubation periods were within the range of their initial velocities at recommended substrate concentrations. In Figure 5, MAOs Michaelis-Menten kinetics curves with and without BNN at an initial rate of velocity (*V*) versus LDS concentration change were illustrated in Lineweaver-Burk plot (Figures 5(a) and 5(b)). All regression lines were crossing the *y*-axes in approximately one point (*V*_max_) while they crossed the *x*-axes at variable *K*_*m*_ values. To confirm the parameters significance of change, MAOs *V*_max_, *K*_*m*_ values changed by BNN were compared to the clinical standard deprenyl (DEP) parameters as a positive control. In Figures 5(c) and 5(d), BNN caused no significant change to maximum velocity (*V*_max_) neither in* h*MAO-A nor in* h*MAO-B. Likewise, DEP showed no change with* h*MAO-A *V*_max_ while 1.3 *μ*M caused an expected significant decrease in DEP* h*MAO-B *V*_max_ (*p* ≤ 0.05). In Figure 5(e), higher BNN concentrations of 18.75, 75, and 300 *μ*M increased* h*MAO-A *K*_*m*_ 2.4-, 4.0-, and 4.8-fold (*p* < 0.0001), with decreased catalytic efficiency to 36, 24, and 20%, respectively (table in Figure 5(e)). BNN highest concentration reduced* h*MAO-A efficiency ratio to be close to DEP 14.85 *μ*M ratio (19%). In other words, BNN* h*MAO-A IC_50_ was able to reduce the efficiency as the DEP* h*MAO-A IC_50_. In Figure 5(f), low concentrations of 10, 20, and 40 *μ*M BNN gradually increased relative* h*MAO-B *K*_*m*_ 7.5-, 12.5-, and 21.5-fold (*p* < 0.0001), respectively. Meanwhile, DEP* h*MAO-B *K*_*m*_ was not significant (0.7 ± 0.5-fold). Calculating relative* h*MAO-B *V*_max_/*K*_*m*_ (table in Figure 5(f)), the catalytic efficiency had been dramatically decreased to only 13, 8, and 6% with increasing BNN. That efficiency decrease was more than DEP at 1.3 *μ*M (26%), meaning that BNN* h*MAO-B IC_50_ was able to reduce the* h*MAO-B efficiency more than 10x DEP IC_50_.

**Figure 6:**
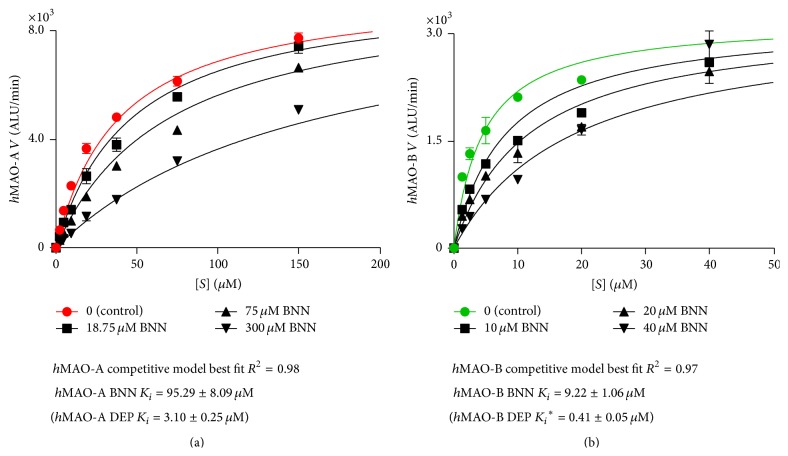
Bavachinin (BNN)* h*MAO-A (a) and* h*MAO-B (b) inhibition best fitted the competitive model of inhibition. Global share fit values of *K*_*i*_ ± SEM, *n* = 3, were calculated by GraphPad Prism and compared to standard selegiline (DEP). All *K*_*i*_ values were calculated using the competitive mode of inhibition, except *K*_*i*_^*∗*^ (noncompetitive inhibition mode) at isozymes initial velocity (*V*) with luciferin derived substrate concentrations ([*S*]).

In Figure 6,* h*MAO-A (Figure 6(a)) and* h*MAO-B (Figure 6(b)) Michaelis-Menten curve with BNN took the best fit to the competitive mode of inhibition using GraphPad Prism 6.02. Alpha mixed mode of inhibition was more than one and very large in both inhibited isozymes. Since *K*_*i*_ determination is independent of substrate, *K*_*i*_ was determined and calculated according to competitive inhibitory mode. BNN* h*MAO-A *K*_*i*_ was higher (95.29 *μ*M, *R* ^2^ = 0.98) than* h*MAO-B (9.22 *μ*M, *R* ^2^ = 0.97) by 10.33-fold. Standard MAO-BI DEP had a lower* h*MAO-A *K*_*i*_ of 3.1 *μ*M (*R* ^2^ = 0.99) for the competitive model than its* h*MAO-B *K*_*i*_ of the noncompetitive model (0.41 *μ*M, *R* ^2^ = 0.98) by 7.56-fold. Thus, BNN SI was equivalent to DEP SI.

### 3.6. Docking Analysis of BNN and BVN into* Human* MAO-A and MAO-B Active Sites

**Figure 7:**
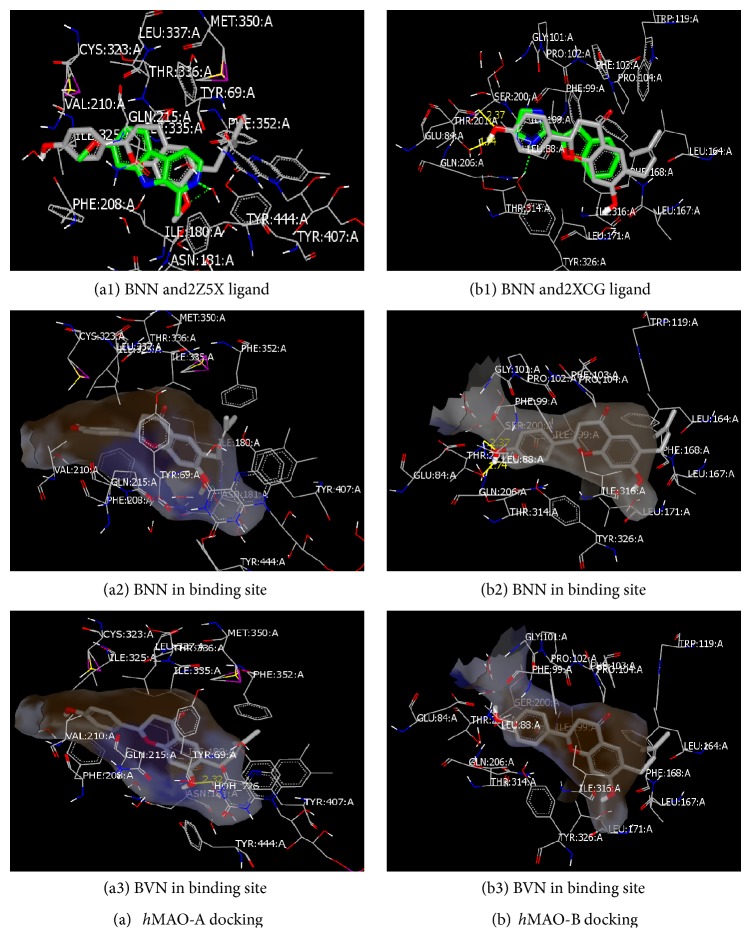
Interaction models of bavachinin (BNN) and bavachin (BVN) in MAO isozymes active sites. Docking poses in (a)* human* MAO-A with BNN and 2Z5X ligand (a1), BNN alone (a2), or BVN alone (a3) and in (b)* human* MAO-B with BNN and 2XCG (b1), BNN alone (b2), or BVN alone (b3). The analogs were surrounded with the closest active site residues where the difference in their interactive behavior with both isozymes is illustrated. Molecular surfaces are highlighted in brown for hydrophobic zones and blue for polar aminoacids.

The highest ranked docking scores and orientation of BNN and BVN at the active sites of each of the* human *MAO-A and MAO-B crystal structures were shown in Table 1 and Figure 7, respectively. In Figure 7, the docking poses represented the possible bioactive conformations of both analogs in the active site of each isozyme in comparison to their cognate ligands (2Z5X and 2XCG). BNN and BVN were predicted to be able to act on the same active site of these ligands. For ease and simplicity, only BNN was shown with the ligands (Figures 7(a1) and 7(b1)). Both BNN and BVN showed similar orientations as their C2-phenoxy group was the closest to entrance cavities of both MAO-A and MAO-B. In MAO-B (Figures 7(b1), 7(b2), and 7(b3)), BNN and BVN chromone core structures were posed in the middle of the hydrophobic surface zone. Their C6-prenyl group was closer to the substrate active site environment, surrounded with leucine, isoleucine, and phenylalanine (LEU: 164: A, ILE: 316: A, and PHE: 168: A). Those highly hydrophobic amino acids zones probably attracted the flavonoid prenyl group. In Figures 7(a1), 7(a2), and 7(a3), where MAO-A has a less lipophilic active site, the chromone structure and the prenyl group seemed to avoid being trapped in the tyrosine sandwich of TYR: 444: A and TYR: 407: A besides FAD that could be important for activity.

In Table 1, the top scoring poses of BNN and BVN were compared to the reversible MAO-BI SAF. In both isozymes, BNN docking scores and interactions were completely different from BVN. BNN MAO-A affinity scores were lower than BVN by more than eightfold to be closer to SAF MAO-A affinity scores. The predicted high affinity of BVN was accompanied by its water-mediated hydrogen- (H-) bonding interaction in the MAO-A active site at (BVN C7-HO…H-OH 726) with 2.32 Å (see also Figure 7(a3)). That was not the case with BNN as C7 is substituted with a methoxy group (see also Figure 7(a2)). No H-bonds were predicted in SAF interaction either. In the MAO-B active site, BNN and SAF showed similar docking scores with the presence of more than one H-bonding interaction. BVN, on the other hand, had lower MAO-B affinity score with no H-bonds observed (see also Figure 7(b3)). BNN was predicted to form two H-bonds with a key amino acid residue threonine (THR: 201: A) with the distances measured from BNN C4′-OH 1.74 and 2.37 Å. SAF was also found to have similar predicted interactions with threonine (1.80 Å) in addition to the other residues. Measured BNN H-bonds distance matched the range of SAF H-bonds.

## 4. Discussion

The current literature suggests that flavonoids are promising candidates as reversible human MAO-A and MAO-B inhibitors [41, 42]. However, there is meager research on the subclass prenylflavanones, possibly due to their very recent isolation from plants. As we continue our research for natural MAO-BIs, two unique prenylflavanones from flavonoids rich PC plant seeds [43] were tested. BNN [35] and BVN [44] (S)-enantiomers (Figure 1) were investigated for their potential to inhibit* h*MAO-A and* h*MAO-B. We showed that PCSEE possesses* h*MAO-B and* h*MAO-A inhibitory effects, while BNN inhibited both isozymes, and BVN activated both isozymes. The obtained results indicated that BNN competitively inhibits both isozymes with more selectivity for* h*MAO-B and very weak inhibitory action for* h*MAO-A. One suggested mechanism of selective inhibition is that by the virtue of BNN C7-methoxy group interactions with C4′-OH group and the key amino acids in* h*MAO-B active site become highly possible.

To investigate PCSEE* h*MAO inhibitory activities and whether BVN and BNN compounds contribute to that activity (Figures 1(i) and 1(ii)), a highly sensitive two-step functional luminescence assay was used. The lower possible interactions with luminescence assay were advantageous with such components for determining inhibitory efficacy, potency, selectivity, and mode of inhibition compared to a spectrophotometric assay. Indeed, the difference between BNN and BVN effects was very clear, showing no BVN inhibitory effects, while BNN inhibited* h*MAO-B more effectively and selectively. For validation and control, our DEP* h*MAO-B inhibition results confirming previous studies used similar luminescence assay and* h*MAOs [45]. Indeed, BNN showed clear selective MAO-B inhibitory effects. In comparison with PCSEE, the BNN* h*MAO-B inhibitory potency was matching PCSEE (BNN IC_50_ of 2.98 ± 0.97 *μ*g/mL compared to PCSEE IC_50_ of 2.25 ± 0.34 *μ*g/mL). Moreover, BNN here is confirmed to be more selective (21.46-fold) than PCSEE (6.29-fold). Thus, BNN can be considered one of the constituents responsible for PCSEE* h*MAO-B potency and relative selectivity.

To investigate whether BNN amount plays an essential role in PCSEE MAO-B inhibition, BNN was identified and quantified in our used extract. The TLC calculation of *R*_*f*_ and the observation of fluorescence colors partially indicate the presence of BNN in PCSEE (Figure 3). In other words, this test provides further evidence of the presence of BNN in PCS. Also, the low *R*_*f*_ value of the matching band of BNN may point out the lipophilic prenylated and methoxy groups in BNN slowed elution with our hydrophilic developing system. PCSEE was previously estimated to contain 2.25 mg/g flavonoids [43] and the flavonoid BNN was reported to be so far only found in PCS methanolic extract (2.16%) and 80% ethanolic extracts (~0.1% w/w) [46, 47]. In our HPLC quantification of BNN in PCSEE, the amount of BNN in the PCSEE dry crude was low (0.21% w/w) but consistently higher than in 80% ethanol PC in water which is possibly due to its low solubility in water. Thus, PCSEE selectivity or potency for MAO inhibition may be caused by other constituents.

In spite of the weak ability of BNN to inhibit MAO-A, it was essential to determine the prenylflavanone mode of inhibition for both isozymes. The double-reciprocal plot, Lineweaver-Burk, for* h*MAO isoforms simply showed BNN to possess a competitive mode of inhibition (Figure 5). The very large alpha values also indicated that the binding of BNN prevents substrate binding to the active site by competitive inhibition and indicates reversible inhibition kinetics [23]. Moreover, each of the* h*MAO isozyme kinetic parameters modulations by BNN (*V*_max_, *K*_*m*_, and *K*_*m*_/*V*_max_) supported the competitive behavior of our compound. From *K*_*m*_/*V*_max_ ratio, BNN reduced the* h*MAO-B catalytic efficiency to be lower than DEP reduction of efficiency in their tested concentrations. In comparing BNN with DEP, BNN potency to inhibit* h*MAO-B was weaker than DEP (*μ*M versus nM range potency). However, BNN IC_50_ was able to reduce* h*MAO-B efficiency ten times more than DEP in concentrations higher than its IC_50_ while it was similar to DEP in their* h*MAO-A IC_50_ values. Also, BNN SI was close to that of DEP (Figure 6). All the above may be summarized in that BNN is weaker MAO-BI than DEP but has equivalent selectivity and the advantages of inhibiting* h*MAO-B competitively and more efficiently than DEP.

We carried out our molecular docking studies to rationalize the structure-activity relationship difference of BNN and BVN toward* h*MAO-A and* h*MAO-B isozymes (Figure 7). Docking studies may give us an insight into how substituting C7-OH group in BVN with a C7-methoxy group makes it an effective target towards* h*MAO-B. BNN was also compared to SAF for its natural chemical source and its selective and reversible MAO-B inhibitory characters. Docking studies analysis of BNN on* human *MAO-A and MAO-B crystal structures predicted highly different affinity behaviors in its binding. Also, BNN showed different affinity from the C7-OH substituted BVN on the same isozyme.

BNN had higher MAO-B docking affinity than MAO-A. One possible explanation of these differences in affinity predictions is the difference between MAO-B and MAO-A isozymes active sites as MAO-B has a largely lipophilic cavity structure [48]. That lipophilic zone might attract and stabilize the highly lipophilic chromone phenyl ring and the C6-prenyl substitution with the less lipophilic MAO-A zone. Synthetic flavanones were reported to have potent MAO-B inhibitory effects in one single impressive study [49] to be superior to their flavone's or thioflavones structures in a low *μ*M range. The most selective flavanone in that study was with the hydrophobic C6-methyl group and C4′-fluorine substitute where MAO-B inhibitory selectivity reached up to 769-fold. This selectivity and potency are consistent with our results and conclusions for the C6 importance in the inhibition. Moreover, C6 lipophilic aromatic substitutions in reported synthetic chromone derivatives also showed highly potent and reversible selective MAO-B inhibition [50]. Thus, lipophilic prenyl group on its C6 position may have played a role in reversibility and selectivity to inhibit MAO-B. However, both active BNN and nonactive BVN have similar C6-prenyl groups in their chromone of the prenylflavanone structures.

MAO-B had an H-bond forming residues on the outer part of its active site [48]. In addition to the prenyl group of the chromone, BNN was predicted to form H-bonding interactions with* h*MAO-B active site residues while BVN was predicted not to form such bonds (Table 1). The more lipophilic BNN C7-methoxy group might be indirectly responsible for conformational changes or more stearic stabilization in the lipophilic pockets of MAO-B active site that led to new H-bond interactions between BNN C4′-OH and the threonine in MAO-B entrance cavity. In comparison to the more hydrophilic BVN C7-OH, this character may have been lost (Figure 7(b3)), explaining the inhibitory selectivity of BNN to be different from MAO-A and from BVN and why BVN lost its MAO-B inhibition. Also, BNN interactions were very close to SAF in both affinity and H-bond formation predictions which indicate possible similar inhibitory behavior as reversible MAO-BIs. Consistently, it was previously reported that C7 hydrophobic substitutes in chromone derivatives showed* h*MAO-B reversible inhibition [51]. Thus, prenylflavanone BNN C4′ hydrophilic and C7 hydrophobic substitution may also contribute to its competitive reversible and selective MAO-B inhibition. This conclusion may be helpful in searching for more prenylflavanones with different lipophilic C7 substitutions for more specific and potent natural or seminatural* h*MAO-BIs.

Pharmacologically, there had been no reports concerning BNN to treat PD. However, it was repeatedly isolated and tested for its anti-inflammatory activities, one of the symptoms of PD and AD. BNN isolated from PCS showed* in vivo* anti-inflammatory activity [52], inhibited IL-6-induced STAT3 inflammation activation [53], inhibited IL-4, and other inflammatory cytokines, by inhibiting T-helper 2 cells differentiation [54]. Interestingly, our inactive analog BVN was also reported to have an anti-inflammatory activity. BVN displayed a potent decrease of neuroinflammatory IL-1 *β*-induced nuclear factor-kappa B (NF-*κ*B) that could benefit reported NF-*κ*B cognitive dysfunctions in PD [55, 56]. That means that the anti-inflammatory activity of these two prenylflavanones was maintained, which is an additional advantage of BNN structure.

From another perspective, BNN was shown to attenuate amyloid-beta A*β*42-induced toxicity in an SH-SY5Y cell model for AD therapy by inhibiting toxic fibrillization and aggregation [46]. Interestingly, DEP neuroprotection in AD was also observed in A*β* peptide A-induced toxicity in the vascular endothelium [57]. Furthermore, BNN was shown to be a potent peroxisome proliferator-activated receptor-*γ* (PPAR-*γ*) agonist [58] which is considered neuroprotective in PD neurodegeneration [59, 60]. Flavanones were also reported to have vasodilation activities [61], an action attributed to DEP benefit in neurodegeneration [57]. In addition, BNN was reported to have high oral absorption [62], and* in silico* investigation predicted high blood brain barrier penetration in humans [46]. Thus, knowing that BNN possesses anti-inflammatory properties and neuroprotective activities besides its selective reversible MAO-B inhibitory activities, BNN may serve as an ideal compound for PD therapy because of its potential multifunctional activities.

In conclusion, the unique prenylflavanone BNN found in PCSEE in low amounts possesses selective and reversible MAO-B inhibition. The biochemical and* in silico* results indicate that BNN inhibitory interactions of MAOs are most certainly through the competitive catalytic site related mechanism. The reported reversible MOA inhibitory effects of BNN could provide safer substitute for the currently used classical MAO-BIs. BNN binding preference to MAO-B may be due to its closer hydrophobic C6-prenyl and C7-methoxy groups and hydrophilic C4′-H-bonding forming interactions altogether. Moreover, BNN C7 may be crucial for the specific reversible inhibitory interaction of MAO-B. The results obtained suggest that BNN could represent a novel class of natural reversible MAO-BIs. The properties of the prenylflavanones BNN may qualify it to be an MOA-B inhibitor with anti-inflammatory agent for the therapeutic management of PD and other neurological disordrer.

## Figures and Tables

**Table 1:** Docking affinity scores and possible H-bond formation by bavachinin (BNN) and bavachin (BVN) in comparison with reversible MAO-BI safinamide (SAF)^*∗*^.

Ligand	MAO-A active site		MAO-B active site					MAO inhibition selectivity
	Docking Score^a^	H-bonds Predicted	Docking Score^a^	H-bonds Predicted	Å	Type^b^	Active site residue	
BNN	−1.06	0	−6.82	2	1.74	OH⋯O	THR: 201: A	B
					2.37	HO⋯HN	THR: 201: A	
								
BVN	−8.72	H_2_O-726	−3.95	0	⋯	⋯	⋯	NA
								
SAF^*∗*^	−0.22	0	−6.12	3	1.77	NH⋯O	GLU: 84: A	B
					1.80	NH⋯O	THR: 201: A	
					2.04	NH⋯O	PRO: 102: A	

^a^HYBRID Chemgauss4 scores: root mean square deviation less than 2 Å.

^b^The type of H-bond between ligand and MAO active site amino acid residue.

^*∗*^Reference [38].

## Abbreviations

2-BFI: 2-(2-Benzofuranyl)-2-imidazoline
AD: Alzheimer's disease
ALU: Arbitrary Light Units
BNN: Bavachinin
BVN: Bavachin
DEP: Deprenyl (selegiline)
EE: Ethanolic extract
FAD: Flavin adenine dinucleotide
HAR: Harmine
HBSS: Hank's Balanced Salt Solution
*h*MAO: * Recombinant human* MAO
*h*MAO-BIs: * Recombinant human* MAO-B inhibitors
H-bond: Hydrogen bond
*K*_*m*_: Michaelis Constant
LDS: Luciferin derivative substrate
MAO: Monoamine oxidase
NF-*κ*B: Nuclear factor-kappa B
PC: * Psoralea corylifolia* L.
PCS: * Psoralea corylifolia* L. seeds
PCSEE: PCS ethanolic extract
PD: Parkinson's disease
PDB: Protein Data Bank
RAS: Rasagiline
RCSB: Research Collaborator for Structural Bioinformatics
RS: Relative selectivity
SAF: Safinamide
SI: Selectivity index
TLC: Thin layer chromatography
*V*_max_: Maximum velocity.
